# *APOE*-by-sex interactions on brain structure and metabolism in healthy elderly controls

**DOI:** 10.18632/oncotarget.5185

**Published:** 2015-09-10

**Authors:** Frederic Sampedro, Eduard Vilaplana, Mony J de Leon, Daniel Alcolea, Jordi Pegueroles, Victor Montal, María Carmona-Iragui, Isabel Sala, María-Belén Sánchez-Saudinos, Sofía Antón-Aguirre, Estrella Morenas-Rodríguez, Valle Camacho, Carles Falcón, Javier Pavía, Domènec Ros, Jordi Clarimón, Rafael Blesa, Alberto Lleó, Juan Fortea, for the Alzheimer’s Disease Neuroimaging Initiative

**Affiliations:** ^1^ Memory Unit, Department of Neurology, Hospital de la Santa Creu i Sant Pau- Biomedical Research Institute Sant Pau- Universitat Autònoma de Barcelona, Barcelona, Spain; ^2^ Centro de Investigación Biomédica en Red de Enfermedades Neurodegenerativas. CIBERNED, Madrid, Spain; ^3^ Nuclear Medicine Department, Hospital de la Santa Creu i Sant Pau- Biomedical Research Institute Sant Pau- Universitat Autònoma de Barcelona, Barcelona, Spain; ^4^ New York University School of Medicine, New York, NY, USA; ^5^ Unitat de Biofísica i Bioenginyeria, Departament de Ciències Fisiològiques I, Facultat de Medicina, Universitat de Barcelona – IDIBAPS, Barcelona, Spain; ^6^ Nuclear Medicine Department. Hospital Clínic de Barcelona, Barcelona, Spain; ^7^ Biomedical Research Networking Center in Bioengineering, Biomaterials and Nanomedicine - CIBER-BBN, Barcelona, Spain

**Keywords:** Gerotarget, Alzheimer’s disease, aging, APOE, MRI, PET-FDG

## Abstract

**Background:**

The *APOE* effect on Alzheimer Disease (AD) risk is stronger in women than in men but its mechanisms have not been established. We assessed the *APOE*-by-sex interaction on core CSF biomarkers, brain metabolism and structure in healthy elderly control individuals (HC).

**Methods:**

Cross-sectional study. HC from the Alzheimer’s Disease Neuroimaging Initiative with available CSF (*n* = 274) and/or 3T-MRI (*n* = 168) and/or a FDG-PET analyses (*n* = 328) were selected. CSF amyloid-β_1–42_ (Aβ_1–42_), total-tau (t-tau) and phospho-tau (p-tau_181p_) levels were measured by Luminex assays. We analyzed the *APOE*-by-sex interaction on the CSF biomarkers in an analysis of covariance (ANCOVA). FDG uptake was analyzed by SPM8 and cortical thickness (CTh) was measured by FreeSurfer. FDG and CTh difference maps were derived from interaction and group analyses.

**Results:**

*APOE4* carriers had lower CSF Aβ_1–42_ and higher CSF p-tau_181p_ values than non-carriers, but there was no *APOE*-by-sex interaction on CSF biomarkers. The *APOE*-by-sex interaction on brain metabolism and brain structure was significant. Sex stratification showed that female *APOE4* carriers presented widespread brain hypometabolism and cortical thinning compared to female non-carriers whereas male *APOE4* carriers showed only a small cluster of hypometabolism and regions of cortical thickening compared to male non-carriers.

**Conclusions:**

The impact of *APOE4* on brain metabolism and structure is modified by sex. Female *APOE4* carriers show greater hypometabolism and atrophy than male carriers. This *APOE*-by-sex interaction should be considered in clinical trials in preclinical AD where *APOE4* status is a selection criterion.

## INTRODUCTION

The apolipoprotein E (*APOE*) genotype is the strongest genetic risk factor for Alzheimer’s disease (AD) [1]. It has three isoforms, ε2, ε3 and ε4. The *APOE* ε4 allele (*APOE4*) increases the risk for AD [2]. The effect of the *APOE4* allele on AD biomarkers in healthy controls (HC) has been widely studied [3], [4]. *APOE4* carriers have consistently lower cerebrospinal fluid (CSF) β-amyloid 1–42 (Aβ_1–42_) levels than non-carriers, but the differences in tau levels are more controversial [5]–[7]. Most, [8]–[10] but not all [18F]-fluorodeoxyglucose (FDG) PET studies [11]–[13] have shown hypometabolism in AD-related regions in *APOE4* carriers in late-middle age [8] and even earlier [10]. A gene-dosage effect on the hypometabolism has also been reported [9]. The relationship between the *APOE* genotype and brain structure is more controversial. Many cross-sectional studies have reported cortical thinning or hippocampal atrophy, [3], [4], [14] while several others have found no relationship [15] and two have reported increased gray matter in relation to the *APOE4* allele [16], [17].

Several factors might account for the conflicting results. First, the age-range differences between studies are critical because distinct effects of *APOE* across the lifespan have been described [18]. Not all brain changes associated with the *APOE* genotype reflect incipient AD. *APOE* has been implicated in normal human brain development [19]. Second, there are amyloid dependent [20] and independent [21] mechanisms underlying the *APOE* influences on AD risk. However, most studies assessing the role of *APOE* on brain structure and metabolism do not assess AD pathophysiological biomarkers to disentangle these mechanisms. Third, *APOE4* is likely to interact with other pathological factors, complicating the isolation of a unique genetic effect [4]. And fourth, some of the inconsistent imaging and biochemical findings related to *APOE* in HC might result from neglecting a possible *APOE*-by-sex interaction [6]. Most studies to date have included sex as a covariate in the analyses but they did not explicitly test for an *APOE*-by-sex interaction.

The finding that the *APOE* effect on AD risk is stronger in women than in men was reported in early studies, [22], [23] confirmed in meta-analyses, [23], [24] and in a recent longitudinal study [6]. However, only two studies have assessed *APOE*-by-sex interactions on AD biomarkers. Altmann et al found a significant interaction for tau in mild cognitive impairment patients [6]. Damoiseaux et al reported a significant *APOE*-by-sex interaction for CSF tau levels and default mode network abnormalities in healthy controls [25].

The interaction between *APOE4* and sex on brain structure and metabolism has not been established. This interaction could affect the design and interpretation of prevention trials in preclinical AD in which APOE is a selection criterion (i.e. the Alzheimer’s Prevention Initiative *APOE4* Trial, NIH project number 1UF1AG046150-01). The aim of the present study was to examine the interactions between *APOE4* and sex on brain metabolism and structure, based on the hypothesis that the *APOE4* allele exerts a differential adverse effect on brain metabolism and structure depending on sex.

## RESULTS

Demographic and clinical of the participants in the CSF, FDG and MRI subsets are summarized separately in the Table 1. CSF was available in 274 HC individuals, 328 had an FDG PET, 225 had a 3T MRI, and 137 subjects had all three biomarkers. There were no significant differences between the MRI, PET and CSF subsets in age, sex, *APOE* status, MMSE or CSF biomarkers. There were no significant differences in age, *APOE* status, MMSE or CSF biomarkers between males and females in all three subsets. In the FDG and CSF subsets, males had higher years of education than females (*p* < 0.001), but in the MRI subset this difference did not reach significance.

**Table 1:** Demographic, cerebrospinal fluid and clinical data in the CSF, FDG-PET and MRI Alzheimer’s Disease Neuroimage Initiative subsets

		MRI (*N* = 168)	FDG-PET (*N* = 328)	CSF (*N* = 274)
***APOE4* N (%)**		50 (29.76%)	87 (26.5%)	71 (25.9%)
**AGE**		73.4 (6.02)	74.5 (5.57)	74.4 (5.97)
**SEX (% Females)**		53.6%	49.4%	50.4%
**MMSE**		29.1 (1.07)	29.0 (1.24)	29.1 (1.15)
**YEARS OF EDUCATION**		16.6 (2.55)	16.3 (2.77)	16.3 (2.69)
Aβ_1–42_***	TOTAL	200.7 (49.92)	201.4 (52.46)	200.6 (52.51)
	*APOE4*−	211.3* (46.32)	213.5* (46.87)	212.1* (47.81)
	*APOE4*+	175.4* (49.58)	165.2* (51.85)	167.9* (51.87)
p-tau_p181_***	TOTAL	32.4 (16.41)	30.78 (18.14)	30.48 (17.97)
	*APOE4*−	31.3 (16.68)	28.3* (15.31)	28.2* (15.23)
	*APOE4*+	35.0 (15.62)	38.1* (23.38)	36.9*(23.10)
t-tau***	TOTAL	66.0 (31.88)	68.9 (34.57)	68.4 (32.12)
	*APOE4*−	65.1 (32.60)	67.0 (34.84)	66.0** (30.29)
	*APOE4*+	68.2 (30.34)	74.5 (33.41)	75.1** (36.22)

*APOE4+* = apolipoprotein E ε4 allele carrier, APOE− = apolipoprotein E ε4 allele non-carrier

Values are expressed as mean (standard deviation) unless specified.

* equals *p* < 0.001 and

** equals *p* < 0.05 for the *APOE4* carriers vs non-carriers comparison within each subset. Note that 137 subjects were included in the three subsets.

*** CSF data only available in 146 subjects in the MRI subset and 242 subjects in the PET subset.

*APOE4* carriers had lower CSF Aβ_1–42_ values than non-carriers in all three subsets (*p* < 0.001). *APOE4* carriers had higher CSF p-tau_181p_ values in the three subsets, but these only reached significance in the FDG and CSF subset which had larger sample sizes (*p* < 0.001 and *p* = 0.004 respectively). *APOE4* carriers had higher CSF t-tau values in the three subsets, but these only reached significance in the CSF subset (*p* < 0.05). There were no significant differences in MMSE scores or education between *APOE4* carriers compared to non-carriers in any of the subsets. There were no significant differences between males and females in CSF biomarkers. Neither was there an *APOE*-by-sex interaction on CSF Aβ_1–42,_ CSF t-tau or CSF p-tau_181p_values in the analysis of covariance (ANCOVA) analyses.

### *APOE*-by-sex interaction on brain metabolism

**Figure 1:**
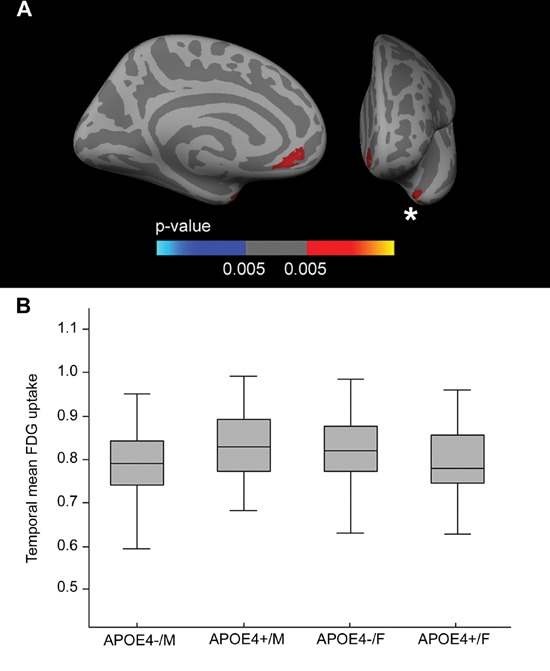
FDG *APOE*-by-sex interaction analysis **A.** Areas in which there is a FDG-uptake interaction between sex and the *APOE4* status (*p* < 0.005 uncorrected) co-varied for age and years of education displayed across the medial and frontal views of the cerebral cortex. **B.** Box and whisker plot illustrating individual FDG-uptake values in the temporal cluster. For each plot, the central black lines show the median value, the regions above and below the black line show the upper and lower quartiles, respectively, and the whiskers extend to the minimum and maximum values. As illustrated, the female *APOE4* carriers showed decreased metabolism in the temporal cortex with respect to female non-carriers. FDG = fluorodeoxyglucose; *APOE* = apolipoprotein E, *APOE4+* = apolipoprotein E ε4 allele carriers, *APOE4−* = apolipoprotein E ε4 allele non-carriers.

Fig. 1A presents this FDG voxel-wise interaction analysis across the cerebral hemispheres, showing voxels with an *APOE*-by-sex interaction, covaried by age and years of education (*p* < 0.005, *k* = 50). Two clusters emerged, one located mainly in the anterior cingulate region and the other in the temporal region. To analyze the directionality, we isolated the temporal cluster, averaged the FDG uptake, and plotted it in box and whisker plots (Fig. 1B). As shown, this interaction was driven by the decreased metabolism in female *APOE4* carriers and the increased metabolism in male *APOE4* carriers. The main and interactive effects of *APOE4* status and sex on brain metabolism in the ANCOVA analysis were significant in the model (interaction term between *APOE4* status and sex: β-coefficient = 0.069, standard error [SE] = 0.021, *p* = 0.001; main effect of *APOE4* status: β-coefficient = −0.037, SE = 0.016, *p* = 0.019; main effect of sex: β-coefficient = −0.041, SE = 0.018, *p* = 0.026). Similar results were found for the anterior cingulate cluster (not shown).

**Figure 2:**
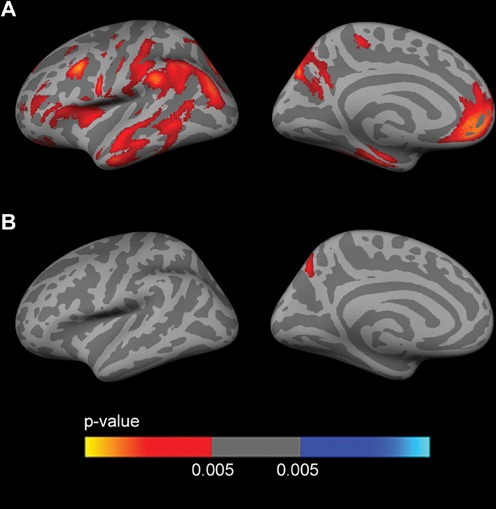
Sex-stratified FDG analyses Analysis between *APOE4* carriers and *APOE4* non-carriers (*p* < 0.005 uncorrected) in **A.** females and **B.** males, co-varied for age and years of education across the lateral and medial views of the cerebral cortex. As shown, female *APOE4* carriers showed widespread clusters of decreased metabolism with respect to female *APOE4* non-carriers (Fig. 2A), whereas male *APOE4* carriers only showed an isolated cluster of decreased metabolism (*p* < 0.005) in the precuneus with respect to male non-carriers (Fig. 2B). FDG = fluorodeoxyglucose; *APOE4* = apolipoprotein E ε4 allele.

Fig. 2 shows the sex stratified *APOE4* group analyses for FDG, covaried by age and years of education. Female *APOE4* carriers showed widespread clusters of decreased metabolism (*p* < 0.005) across the whole cerebral cortex in both hemispheres with respect to *APOE4* non-carriers (Fig. 2A). Male *APOE4* carriers showed an isolated cluster of decreased metabolism (*p* < 0.005) in the precuneus with respect to non-carriers (Fig. 2B).

**Figure 3:**
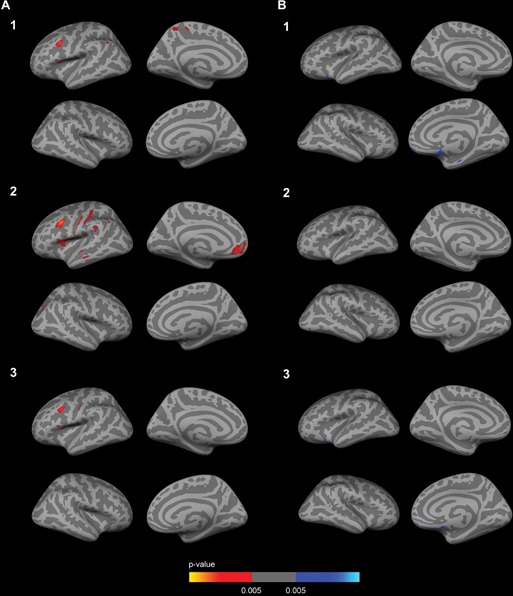
Sex-stratified FDG analyses with CSF biomarker levels included as a covariate Row 1. CSF Aβ_1–42_ levels; Row 2. CSF p-tau_181p_ levels; Row 3 CSF Aβ_1–42_ and p-tau_181p_ levels. The analysis between female *APOE4* carriers and female *APOE4* non-carriers **A1-A3.** showed several clusters of decreased metabolism (*p* < 0.005 uncorrected) co-varied for age. As illustrated, female *APOE4* carriers showed decreased metabolism in the anterior cingulate cortex with respect to female non-carriers after the inclusion of the CSF biomarkers as a covariate. The analysis between male *APOE4* carriers and male *APOE4* non-carriers **B1-B3.** showed several clusters of increased metabolism (*p* < 0.005 uncorrected) co-varied for age. As illustrated, male *APOE4* carriers showed increased metabolism in several clusters in the dorsolateral prefrontal cortex with respect to male *APOE4* non-carriers after the inclusion of CSF Aβ_1–42_ levels or both CSF Aβ_1–42_ and CSF p-tau_181p_ as a covariate (B1 and B3), but not after the inclusion of the CSF p-tau_181p_ levels alone (B2). FDG = fluorodeoxyglucose; *APOE* = apolipoprotein E, *APOE4*: apolipoprotein E ε4 allele

To examine the impact of CSF biomarkers in the *APOE*-by-sex interaction on brain metabolism, we included CSF Aβ_1–42_ and CSF p-tau_181p_ as covariates in the analyses. The inclusion of the CSF biomarkers did not significantly alter the results of the *APOE*-by-sex interaction analysis (not shown) nor the female *APOE4* carriers vs non-carriers comparison (Fig. 3A1–3A3). In the male APOE4 carriers vs non-carriers comparison two clusters of increased metabolism emerged in *APOE4* carriers with respect to male non-carriers in prefrontal regions and a cluster in the medial temporal region when CSF Aβ_1–42_ levels or both Aβ_1–42_ and CSF p-tau_181p_ levels (but not CSF p-tau_181p_ levels alone, Fig. 3B2) were included as a covariate (Fig. 3B1 and 3B3).

### *APOE*-by-sex interaction on brain structure

**Figure 4:**
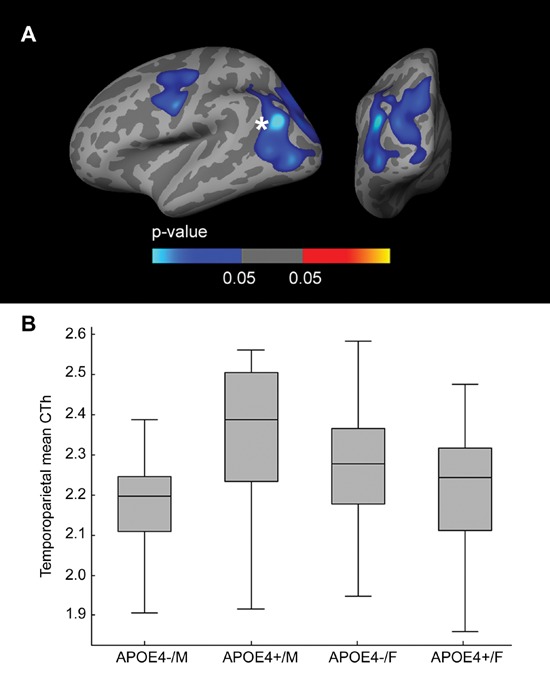
CTh *APOE*-by-Sex interaction analysis **A.** Family-wise corrected (*p* < 0.05) clusters with an interaction between sex and the dichotomized *APOE4* genotype co-varied for age and years of education displayed across the lateral and posterior views of the cerebral cortex. **B.** Box and whisker plot illustrating individual CTh values in the temporo-parietal and occipital cluster. For each plot, the central black lines show the median value, regions above and below the black line show the upper and lower quartiles, respectively, and the whiskers extend to the minimum and maximum values. As illustrated, male *APOE4* carriers showed increased CTh in the temporo-parietal and occipital cluster. CTh = cortical thickness; *APOE* = apolipoprotein E, *APOE4+* = apolipoprotein E ε4 allele carriers, *APOE4−* = apolipoprotein E ε4 allele non-carriers.

Fig. 4A presents the vertex-wise interaction analysis across the whole cortical mantle, covaried by age and years of education, showing voxels with an *APOE*-by-sex interaction. Two large clusters (Family-wise error corrected [FWE] *p* < 0.05) emerged, one in the dorsolateral frontal region and one in the temporoparietal region. To analyze the directionality, we then isolated the temporoparietal cluster, averaged the cortical thickness (CTh), and plotted it in a box and whisker plot (Fig. 4B). As shown, this interaction was mainly driven by the increased CTh in male *APOE4* carriers. The main effects and the interactive effects of *APOE4* status and sex in the ANCOVA analysis were significant in the model (interaction term between *APOE4* status and sex: β-coefficient = −0.228, SE = 0.045, *p* < 0.001; main effect of sex: β-coefficient = 0.149, SE = 0.039, *p* < 0.001; main effect of *APOE4* status: β-coefficient = 0.062, SE = 0.030, *p* = 0.041). Similar results were found for the remaining cluster (not shown).

**Figure 5:**
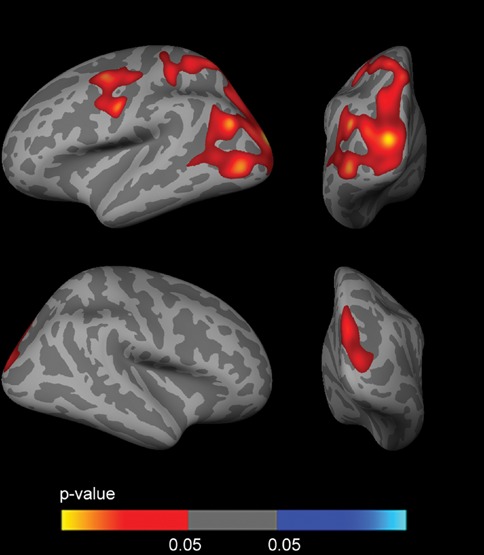
Sex-stratified CTh analyses Analysis between male *APOE4* carriers and male *APOE4* non-carriers, co-varied for age and years of education. As shown, male *APOE4* carriers presented large clusters of increased CTh (FWE *p* < 0.05) in temporo-parieto-occipital regions, mainly in the left hemisphere. The analysis between female *APOE4* carriers and female *APOE4* non-carriers showed clusters of decreased CTh which did not survive FWE correction (not shown). CTh = cortical thickness; *APOE* = apolipoprotein E; FWE = family-wise error corrected (*p* < 0.05).

Fig. 5 shows the sex-stratified *APOE4* CTh analyses, covaried by age and years of education. Male *APOE4* carriers showed 3 large clusters (FWE corrected) of increased CTh with respect to non-carriers. Two of the clusters were observed in the left hemisphere, one in the dorsolateral frontal region and another in the temporoparietal, occipital and precuneus regions. The third cluster was observed in the right hemisphere in the parietal and occipital regions. Female *APOE4* carriers showed cortical thinning in several regions than female *APOE4* non-carriers (not shown as this analysis did not survive FWE correction).

**Figure 6:**
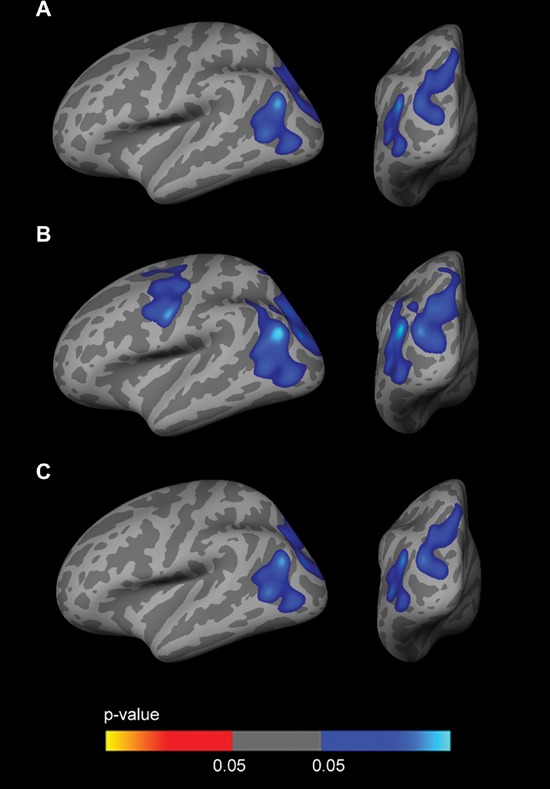
CTh *APOE*-by-Sex interaction analysis with CSF biomarker levels included as covariates Family-wise corrected (*p* < 0.05) clusters with an interaction between sex and the dichotomized *APOE4* genotype co-varied for age and: **A.** CSF Aβ_1–42_ levels; **B.** CSF p-tau_181p_ levels; **C.** CSF Aβ_1–42_ and p-tau_181p_ levels. As illustrated, the inclusion of CSF Aβ_1–42_ levels as a covariate significantly diminished the clusters showing a CTh *APOE*-by-sex interaction. CTh = cortical thickness; *APOE* = apolipoprotein E.

**Figure 7:**
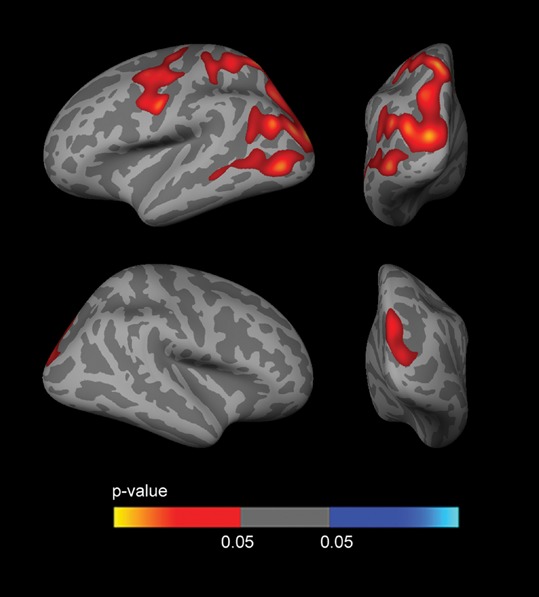
Sex stratified CTh analyses with CSF biomarker levels included as a covariate The analysis between male *APOE4* carriers and male *APOE4* non-carriers showed several clusters of increased CTh (*p* < 0.005 uncorrected) co-varied for age and CSF p-tau_181p_ levels. There were no significant clusters of increased CTh male *APOE4* carriers vs male *APOE4* non-carriers after the inclusion of CSF Aβ_1–42_ levels as a covariate. CTh = cortical thickness; *APOE* = apolipoprotein E.

To examine the influence of CSF biomarkers on the *APOE*-by-sex interaction on brain structure, we included CSF Aβ_1–42_ and CSF p-tau_181p_ as covariates in the analyses. The vertex-wise *APOE*-by-sex interaction analysis across the whole cortical mantle showed a reduction in the significance maps when including CSF biomarkers as covariates, especially Aβ_1–42_ (Fig. 6). In the sex-stratified *APOE4* CTh analyses, the clusters of increased CTh in male *APOE4* carriers disappeared when CSF Aβ_1–42_ levels (but not CSF p-tau_181p_ levels) were included as a covariate (Fig. 7). No result survived FWE correction in females.

All analyses were repeated excluding APOE ε2 allele carriers and including CSF t-tau as a covariate. We also restricted the analyses to non-hispanic white subjects (not shown). The results were not significantly altered in any case.

## DISCUSSION

This study shows for the first time that the impact of the *APOE4* genotype on brain structure and metabolism is modified by sex. We found a significant *APOE*-by-sex interaction on brain metabolism and structure. Female *APOE4* carriers showed brain hypometabolism and cortical thinning with respect to female non-carriers whereas male *APOE4* carriers showed only a small cluster of hypometabolism and cortical thickening with respect to male non-carriers. CSF core AD biomarkers had an influence on brain structural results (and to a lesser extent on brain metabolism).

Epidemiologically, there is strong evidence that supports the *APOE*-by-sex interaction [6], [11], [23]. The only study assessing the *APOE*-by-sex interactions on MRI demonstrated the interaction on resting state functional connectivity but not on gray matter volume [25]. Our results expand these findings. We show an *APOE*-by-sex interaction on both brain structure and metabolism. The discrepancy on brain structure could be due to the differences in the subject population or technical differences (CTh analyses vs voxel-based morphometry [26]). Our FDG results are congruent with those of the aforementioned resting state functional connectivity analyses. *APOE* appears to affect brain network activity which is closely related to neuroenergetic functions [27].

Our metabolic findings suggest that women are metabolically more susceptible to the *APOE4* genotype. Neglecting a possible *APOE*-by-sex interaction on brain metabolism could be one of the reasons for the discordant FDG results [8]–[13]. Male *APOE4* carriers showed increased CTh and females decreased CTh. The finding of cortical thickening in AD vulnerable areas in middle aged (48–75 years old) *APOE4* carriers with respect to non-carriers has already been described [16], [17], but it is in contrast with other works assessing older cohorts [3], [4], [14], [15].

The discrepancies on brain structure might be conciliated if we consider a 2-phase phenomenon model in preclinical AD [28]. In this framework, pathological cortical thickening associated with low CSF Aβ_1–42_ would be followed by atrophy once CSF p-tau_181p_ becomes abnormal [28]. Accordingly, our study shows that the clusters of increased CTh in male *APOE4* carriers disappear when we included CSF Aβ_1–42_ as a covariate. The hypometabolism in female *APOE4* carriers did not disappear when CSF Aβ_1–42_ levels were included as a covariate. The *APOE4* genotype might therefore exert its effects on brain glucose metabolism—at least in part—independently of amyloidogenic pathways [29]. Of note, the inclusion of CSF Aβ_1–42_ levels as a covariate prompted the emergence of several areas of increased metabolism in male *APOE4* carriers. Increased brain metabolism in relation to brain amyloidosis has been previously described [30].

Altogether, our findings support that the mechanisms underlying the increased AD risk in female *APOE4* carriers might occur downstream of Aβ pathology [6]. The *APOE4* effect on lowering CSF Aβ_1–42_ levels is marked in both men and women (with no sex differences) and was also found in our work [6], [25]. The impact of an *APOE*-by-sex interaction on CSF has only been assessed twice and, as in the present work, always with data from the ADNI study. The absence of an *APOE*- by-sex interaction on CSF Aβ_1–42_ levels is in agreement with the two previous works [6], [25]. The impact on CSF p-tau_181p_ levels is less clear. We did not find an *APOE*- by-sex interaction on CSF p-tau_181p_ levels. Such an interaction was reported initially [25] in HC but was not confirmed in the later work with a larger sample size [6]. Nonetheless, this last work did find the interaction for CSF p-tau_181p_ levels in mild cognitive impairment patients. Women, moreover, would be more susceptible and would present more abnormal neuronal injury biomarkers [25] and faster clinical decline [6]. Accordingly, female *APOE4* carriers showed hypometabolism and cortical thinning with respect to non-carriers, suggesting that female *APOE4* carriers might be more advanced in the aforementioned 2-phase phenomenon model in preclinical AD [28].

The mechanisms by which the *APOE* allele modifies the risk for AD have been extensively studied but are not completely understood. Both β-amyloid-dependent [20] and β-amyloid-independent [21] mechanisms have been described. *APOE* appears to affect brain network activity and neuroenergetic functions [27] and to increase microglia reactivity at Aβ plaques in mouse models [31], [32]. These metabolic and inflammatory responses in relation to the *APOE* genotype might differ in males and females, accounting for the differences found.

This work has potential clinical implications. Clinical trials in preclinical AD in which *APOE4* status is a selection criterion are underway (Alzheimer’s Prevention Initiative *APOE4* Trial, NIH project number 1UF1AG046150-01). Our results emphasize the importance of sex stratification when considering the AD risk and its impact on AD topographical biomarkers [33] conferred by the *APOE* genotype. More broadly, the present work stresses the need to consider interactions between biomarkers and risk factors in the AD preclinical phase [28].

The strengths of this study are the inclusion of a relatively high number of subjects and the fact that the results were found in two different topographical AD biomarkers, [34] with congruent findings between the two. The study has some limitations. It is cross-sectional and the age-range sampled does not include young HC to assess the age-range in which amyloid is starting to deposit in the brain of *APOE4* carriers [35].

In conclusion, the impact of *APOE4* on brain structure and metabolism is modified by sex in HC. This interaction should be considered in current clinical trials in preclinical AD in which *APOE4* status is a selection criterion.

## MATERIALS AND METHODS

### Study participants and clinical classification

Data used in the preparation of this article were obtained from the Alzheimer’s Disease Neuroimaging Initiative (ADNI) database (http://adni.loni.usc.edu). The ADNI was launched in 2003 by the National Institute on Aging (NIA), the National Institute of Biomedical Imaging and Bioengineering (NIBIB), the Food and Drug Administration (FDA), private pharmaceutical companies and non-profit organizations, as a $60 million, 5-year public-private partnership. The primary goal of ADNI has been to test whether serial magnetic resonance imaging (MRI), positron emission tomography (PET), other biological markers, and clinical and neuropsychological assessment can be combined to measure the progression of mild cognitive impairment (MCI) and early AD. Determination of sensitive and specific markers of very early AD progression is intended to aid researchers and clinicians to develop new treatments and monitor their effectiveness, as well as lessen the time and cost of clinical trials.

The Principal Investigator of this initiative is Michael W. Weiner, MD, VA Medical Center and University of California – San Francisco. ADNI is the result of efforts of many co-investigators from a broad range of academic institutions and private corporations, and subjects have been recruited from over 50 sites across the U.S. and Canada. The initial goal of ADNI was to recruit 800 subjects but ADNI has been followed by ADNI-GO and ADNI-2. To date these three protocols have recruited over 1500 adults, ages 55 to 90, to participate in the research, consisting of cognitively normal older individuals (HC), people with early or late MCI, and people with early AD. The follow up duration of each group is specified in the protocols for ADNI-1, ADNI-2 and ADNI-GO. Subjects originally recruited for ADNI-1 and ADNI-GO had the option to be followed in ADNI-2. For up-to-date information, see http://www.adni-info.org.

We included all HC with available CSF and/or a 3T-MRI and/or an FDG PET.

### CSF analyses

#### ADNI procedure

Methods for CSF acquisition and biomarker measurement using the ADNI cohort have been reported previously [36]. Aβ_1–42_, total tau (t-tau) and phospho-tau (p-tau_181p_) levels were measured using the multiplex xMAP Luminex platform (Luminex) with Innogenetics (INNO-BIA AlzBio3) immunoassay kit–based reagents.

### MRI and FDG-PET imaging procedures

#### ADNI acquisition procedure

The details of MRI and FDG-PET acquisition are available elsewhere (http://www.adni-info.org).

#### FDG-PET processing procedure

FDG-PET images were downloaded in the most processed format. They were intensity-scaled by the reference pons-vermis region [37], spatially normalized using SPM8 [http://www.fil.ion.ucl.ac.uk/spm/] to the Montreal Neurological Institute (MNI) PET template and spatially smoothed with a Gaussian kernel of full width at half-maximum (FWHM) of 8 mm. All resulting images were visually inspected to check for possible registration errors. Voxel-wise results were displayed at *p* < 0.005 (uncorrected) using an extent threshold *k* = 50, and projected on an inflated single-subject cortical surface reconstruction.

#### Cortical thickness processing procedure

Cortical reconstruction of the structural images was performed with the FreeSurfer software package, version 5.1 (http://surfer.nmr.mgh.harvard.edu). The procedures have been fully described elsewhere [38]. Estimated surfaces were inspected to detect errors in the automatic segmentation procedure. Fifty-seven of the 225 N3 processed MRI analyzed were excluded because of segmentation errors and 168 were included in the analyses. A Gaussian kernel of 15 mm full-width at half maximum was applied. To avoid false positives, we tested Monte Carlo simulation with 10,000 repeats in Qdec (family-wise error [FWE], *p* < 0.05). Only regions that survived FWE are presented in the figures.

### Statistical methods

Group analyses were made using SPSS (SPSS Inc, Chicago, IL). Comparisons between groups were performed using the two-tailed Student *t* test for continuous variables and a chi-square test for categorical variables.

The main objective of our work was to study the *APOE*-by-sex interaction on brain metabolism and brain structure. Two approaches were used: interaction and sex-stratified analyses. We carried out an ANCOVA as implemented in SPM and FreeSurfer for the PET and MRI analyses, respectively, using the *APOE* genotype (*APOE4* carrier vs *APOE4* non-carrier) and sex as binary categorical independent variables, and age and years of education as variables of no interest to assess the interaction.

To examine the impact of CSF biomarkers on the FDG PET and CTh analyses, we introduced CSF biomarkers as covariates in the analyses. All analyses were repeated excluding *APOE2* carriers and restricting to only non-hispanic white subjects.

Clusters derived from the interaction analyses in FDG or CTh were isolated to analyze the directionality of the interactive effects for each variable within an ANCOVA model, using age as a covariate. Specifically, we used the following model for FDG-PET and MRI:

Mean cluster FDG uptake (or mean cluster CTh) = â_0_ + â_1_*SEX + â_2_*APOE + â_3_*[SEX*APOE] + age

The same ANCOVA approach was used for the CSF analyses to test for an interactive effect of *APOE* genotype and sex in CSF biomarker levels.
